# Unveiling the impact of colonic pH and pH-sensing receptors in blood pressure regulation

**DOI:** 10.1080/19490976.2026.2656346

**Published:** 2026-04-11

**Authors:** Evany Dinakis, Liang Xie, Elizabeth A. Vecchio, Charles R. Mackay, Francine Z. Marques

**Affiliations:** a Department of Pharmacology, Hypertension Research Laboratory, Biomedical Discovery Institute, Faculty of Medicine, Nursing and Health Sciences, Monash University, Clayton, Australia; b Victorian Heart Institute, Monash University, Melbourne, Australia; c Department of Obstetrics & Gynaecology, Precision Medicine Translational Research Programme, Yong Loo Lin School of Medicine, National University of Singapore, Singapore, Singapore; d School of Pharmaceutical Sciences, Shandong Analysis and Test Center, Qilu University of Technology (Shandong Academy of Sciences), Jinan, China; e Baker Heart and Diabetes Institute, Melbourne, Australia

**Keywords:** pH, microbiota, blood pressure, short-chain fatty acids, G protein-coupled receptors

## Abstract

Colonic luminal pH is a fundamental feature of the gut environment, shaped largely by the composition and activity of the gut microbiota. Diets rich in fermentable fiber lower the colonic pH primarily through their microbial fermentation, which produces short-chain fatty acids (SCFAs) as metabolic by-products. While the local effects of colonic pH on microbiota composition and intestinal function are increasingly well defined, its systemic consequences remain poorly understood. This review explores the determinants of colonic pH and its dynamic interactions with the gut microbiota, with a particular focus on how these processes may influence and be influenced by host physiology beyond the gastrointestinal (GI) tract. Since dietary fiber and its acidic metabolites confer protection against hypertension, there is growing interest in whether fiber-induced colonic acidification contributes to blood pressure regulation. Notably, participants with hypertension exhibit a higher (more alkaline) colonic minimum pH compared to those with normal blood pressure, further supporting the potential link between luminal acidity and blood pressure control. Particular attention has been given to proton-sensing G protein-coupled receptors (GPCRs), namely, GPR4, GPR65 and GPR68, which are increasingly implicated in regulating immune and cardiovascular functions. Emerging evidence suggests that gene–environment interactions involving GPR65 and GPR68 may influence blood pressure regulation through pH-sensitive pathways; GPR65 primarily via immune modulation, and GPR68 through vascular mechanisms. Therefore, understanding how colonic pH impacts these pathways may uncover novel therapeutic targets for hypertension.

## Introduction

High blood pressure (BP), or hypertension, is defined as persistently elevated systolic BP > 140 mmHg and/or diastolic BP >90 mmHg.^1^ Hypertension is the leading cause of non-communicable diseases and mortality,^2-4^ affecting more than one billion people worldwide.^5^ Despite it being a modifiable risk factor, the majority of patients with hypertension have suboptimal or poorly managed BP.^6^ This is likely attributed to underdiagnosis and/or subpar management, given that only approximately 50% of diagnosed individuals receive treatment, and of those, just one-third achieve adequate BP control.^7^ Consequently, uncontrolled high BP contributes to the onset of cardiovascular disease (CVD), including heart disease, chronic kidney disease, and stroke.^8-11^ Therefore, it is imperative to understand how BP is regulated to achieve better BP control.

Hypertension has a complex etiology, a concept coined as the ‘Mosaic Theory of Hypertension’ proposed by Dr Irvine Page over 60 y ago.^12^ This principle has prevailed to the present day; however, it has been modified over the years as our understanding of BP regulation has evolved.^13^^,^^14^ The 2021 revised version of Page's Mosaic Theory expands on the original concept between vascular, neural, and chemical interactions by integrating cellular (i.e., vascular dysfunction, the immune and central nervous systems, and renal function) and genetic mechanisms contributing to the pathogenesis of hypertension.^14^ Notably, it now also includes the microbiome.^14^ The largest number of microbes in the body is localized in the large intestine,^15^ commonly called the gut microbiome. The gut microbiome not only refers to the microbial community and its genetic material, but also its structural elements, functional potential, metabolites, and surrounding environmental conditions.^16-18^ In contrast, the gut microbiota refers to the community of living microorganisms, including bacteria, archaea, viruses, and fungi, that reside in the gut.^17^^,^^19^^,^^20^ Notably, the vast majority of the gut microbiota is comprised of bacteria.^15^^,^^17^

Numerous studies have implicated alterations in the gut microbiota (i.e., gut dysbiosis) in the development and progression of hypertension.^21-24^ It has been well-characterized that hypertensive patients typically exhibit reduced microbial richness and diversity, often accompanied by an overrepresentation of gram-negative bacteria (e.g., *Klebsiella, Parabacteroides*, *Prevotella*).^22^^,^^25-28^ Moreover, germ-free (GF) mice, which lack any microbiome, have served as a powerful and widely used tool to examine the effects of the microbiome in many disease models, including hypertension.^29^ A pivotal study demonstrated that GF mice subjected to fecal microbiota transplantation (FMT) from two hypertensive patients developed higher systolic and diastolic BP than recipient GF mice of a normotensive participant.^22^ However, these findings were not replicated in a randomized clinical trial involving 126 participants, who were followed for 90 d after FMT.^30^ Thus, while the current body of evidence implicates the gut microbiota as a contributing factor in BP outcomes, the relationship between the two remains unclear, with ongoing debate over whether it is causal or merely associative.

**Figure 1.**
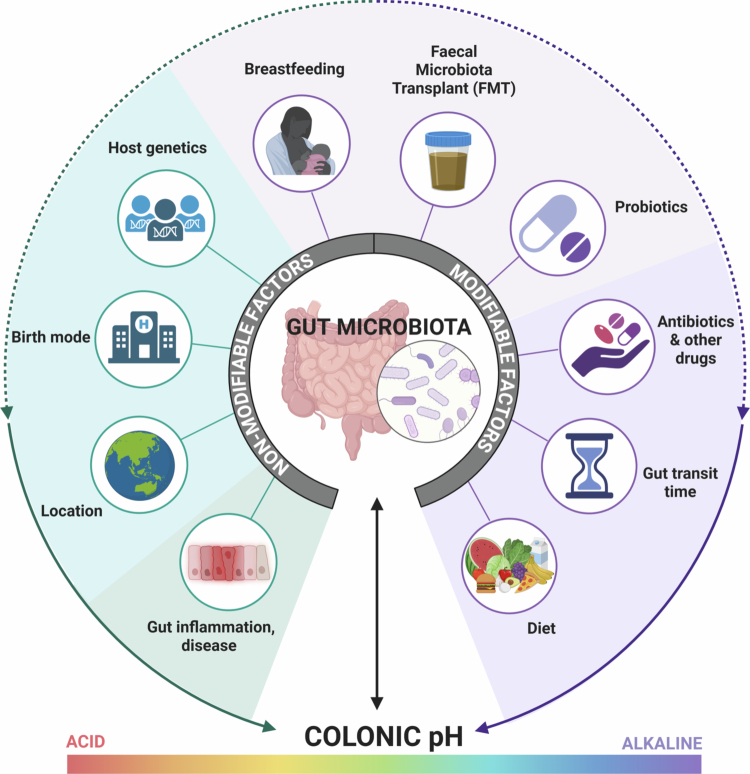
A suite of factors, both nonmodifiable and modifiable, influences the composition, relative abundance and metabolic activity of the gut microbiota, which collectively shape host‒microbiota interactions and downstream physiological outcomes, including the modulation of the colonic pH. Nonmodifiable factors such as host genetics, birth mode, and geographic location influence the gut microbiota composition and function. However, it remains unclear how these factors may also contribute to colonic pH regulation, either independently or through their effects on the gut microbiota. Current evidence suggests that certain predispositions to gut-related inflammatory conditions and diseases, along with variations in gut transit time, are key contributors to colonic pH. Similarly, modifiable lifestyle factors, including antibiotic use, medication consumption and diet (see Figure 2), have been well characterized as key modulators of the gut microbiota. Consequently, their impact on colonic pH has also been reported, although these effects are highly context dependent. Other modifiable factors, such as breastfeeding, fecal microbiota transplantation (FMT), and probiotic consumption, and their impact on colonic pH remain largely unexplored. Figure created with Biorender.com.

Lifestyle modifications are the first-line therapy for preventing, managing, and treating hypertension.^1^^,^^31^ Likewise, the composition of the gut microbiota is strongly influenced by a suite of factors, including host genetics, acquisition at birth, use of antibiotics and other medications, and importantly, diet (Figure 1).^32-36^ Inadequate dietary habits, particularly low consumption of high-fiber foods such as whole grains, vegetables, and fruits, are a key modifiable risk factor for hypertension and CVD.^4^^,^^37^ Importantly, increased fiber intake is associated with a 15%−30% reduction in CVD mortality.^38^ Because vertebrates are not enzymatically equipped to digest these fibers, they reach the large intestine intact, where commensal bacteria residing in the colon have more than 1000 enzymes to aid in the fermentation of certain fibers such as resistant starches.^39^ This leads to the production of metabolites called short-chain fatty acids (SCFAs), particularly acetate, propionate, and butyrate.^40-42^ Studies using pre-clinical models of hypertension have demonstrated that SCFAs play a foundational role in the cardioprotective effects and associated modulation of the gut microbiota mediated by dietary fiber.^28^^,^^43-45^ Moreover, a randomized clinical trial showed that an intervention with a fermentable fiber enriched with SCFAs, delivered to the large intestine, reduced 24-h systolic BP in patients untreated for hypertension.^46^

Importantly, SCFAs are acidic, and their accumulation following fiber fermentation results in significantly lower (i.e., more acidic) colonic luminal and interstitial fluid pH.^47^ Gut pH is critical for shaping the gut microbiota, as microbial species thrive in specific pH ranges.^48^ A more acidic gut environment, such as that produced by SCFAs during fiber fermentation, can promote the growth of beneficial bacteria and inhibit the growth of harmful pathogens.^49^^,^^50^ Therefore, the dynamic interaction between gut pH and gut microbiota composition is essential for maintaining gut health (Figure 1). However, this relationship extends beyond the gut itself and influences systemic outcomes.^51-55^ Thus, while the role of dietary fiber and microbial metabolites in cardiovascular health has been increasingly recognized, the importance of the gut pH in BP regulation remains an emerging area. In this Review, we explore how localized changes in colonic pH—where fiber fermentation occurs—may influence host cardiovascular outcomes by altering microbial composition and systemic responses following the activation of pH-sensitive mechanisms in the context of hypertension.

## Physiological pH landscapes of the host

pH regulation is essential for cellular, tissue, and systemic function.^56-58^ While the cytosolic environment is typically maintained at a neutral pH of 7.0–7.2, intracellular organelles exhibit distinct pH conditions suited to their specialized roles.^59^ For example, lysosomes function optimally in an acidic pH range between 4.5 and 4.7 to support enzymatic degradation and catabolism.^60^ In contrast, mitochondria sustain a transmembrane pH gradient between pH 7-8, crucial for adenosine triphosphate (ATP) synthesis.^61^ Blood pH is tightly maintained between 7.35 and 7.45.^62^ This homeostasis is maintained primarily by the pulmonary and renal systems; the pulmonary system compensates for pH fluctuations through the expiration of carbon dioxide, while the renal system excretes or reabsorbs acid-base equivalents to stabilize systemic pH.^58^ For example, the endogenous metabolism of a Western diet, which typically includes larger intakes of animal protein and phospholipids, generates non-volatile acids such as phosphoric acid and sulfuric acid.^63^ To maintain acid-base homeostasis, these non-volatile acids are excreted by the kidneys.^58^ In individual cell types and whole organs, pH largely depends on the interstitial fluid surrounding the cells, and can vary substantially to support specialized tissue function.^64^ For example, skin maintains a highly acidic pH (i.e., between pH 4–5), which is a critical antimicrobial barrier.^65^ In contrast, a more alkaline pH (greater than or equal to 7.2) supports osteoclast remodeling and osteoblast proliferation in bones.^66^^,^^67^

### Gut and colonic pH

**Figure 2.**
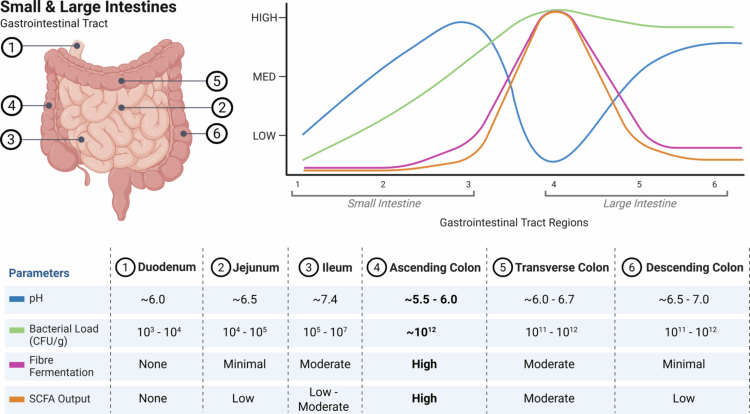
Regional variations in host and environmental parameters along the gastrointestinal (GI) tract, specifically the small and large intestines. pH (blue) and bacterial load (colony-forming units [CFU]/gram; green) follow a steady incline along the three major regions of the small intestine: the duodenum, jejunum, and ileum, respectively. Fermentable fiber bypasses fermentation in the small intestine and reaches the large intestine intact; therefore, fiber fermentation (pink) and subsequent short-chain fatty acid (SCFA) production (orange), which is the primary metabolic output, remain at low levels. All the parameters peak in the ascending colon (bold), with the exception of pH, which reaches its lowest (most acidic) point and progressively plateaus throughout the transverse and descending colon accordingly. Figure created with Biorender.com.

The gastrointestinal (GI) tract is among the most critical physiological systems that depend on the maintenance of an acidic milieu.^68^ The pH of the GI tract can be determined in humans using radiotelemetric devices, such as wireless motility capsules.^69-74^ They have proven to be an indispensable tool in the ongoing effort to map and characterize pH dynamics across different regions of the human GI tract (Figure 2). The first reported study detailing GI pH measurements using a radiotelemetry capsule was performed in 2 healthy subjects and 7 patients with miscellaneous GI disorders in 1972.^69^ In addition to the limited sample size, a major caveat of the study was the 1-h sampling interval between consecutive pH measurements,^69^ which likely resulted in the loss of important fluctuations in luminal pH dynamics. A more robust pH profile of the human gut lumen, including colon sections, was later documented in 1988 in 72 healthy participants.^51^ Importantly, the sampling interval between pH measurements was 12 s.^51^ The average luminal pH within the proximal small intestine was reported to be 6.4.^51^ The mean luminal pH of the colon was reportedly ~6.5, with the proximal colon at pH 6.2 and the distal colon at 7.3.^51^ More recently, studies employing advanced capsules such as the SmartPill and IntelliCap have provided real-time, continuous measurements of luminal pH in the small and large intestines, with sampling intervals ranging from 5 to 10 s, as reviewed in detail elsewhere.^75^ Reports consistently reflect a gradual rise in pH through the small intestine, starting at approximately 6.6 in the proximal small intestine and rising to ~7.5 distally; the proximal colon generally maintains a more acidic environment (pH ~5.5–6.0), whereas the distal colon typically presents with a slightly more alkaline pH (~6.5–7.0).^73^^,^^74^^,^^76-79^ Nonetheless, substantial regional variation in luminal pH is observed along the GI tract, reflecting its compartmentalized physiological functions.

### Colonic pH and the gut microbiota

The GI tract harbors the largest and most diverse microbiota community in the body.^15^ However, the composition and abundance of commensal bacteria within different regions of the GI tract of an adult are not homogenous.^80-82^ Microbial abundance is relatively low in the small intestine. Yet, the abundance of microorganisms within the colon increases to 10^12^ microorganisms/ml,^83^^,^^84^ making it the most densely populated and key site for host‒microbiota interactions within the GI tract (Figure 2). Colonic luminal pH strongly influences the composition and activity of the gut microbiota.^49^^,^^85^ In turn, colonic pH is modulated indirectly by the gut microbiota, primarily through their metabolic activity,^86-88^ creating a feedback loop between microbial metabolism and the colonic luminal pH. Therefore, it can be argued that the relationship between colonic pH and the gut microbiota is mutually exclusive.

Numerous murine studies have demonstrated that the colonic lumen's acidity depends on the presence of the gut microbiota. GF mice, which lack microbial colonization of the colon, exhibit a more alkaline colonic pH (between 7.72 and 8.14).^89^ Treatment with a cocktail of broad-spectrum antibiotics (e.g., ampicillin, cefoperazone, and vancomycin), which indiscriminately depletes the overall bacterial load in the colon, results in colonic pH alkalization similar to that observed in GF mice.^89^ In contrast, acidic pH homeostasis is necessary within the colon to maintain a hospitable environment for the colonization of gut bacteria.^89^ Interestingly, it was not until the 21st century that pH became recognized as a key factor influencing gut microbiota dynamics. The first report, published in 2002, demonstrated that pH influences the bacterial composition of *in vitro* cultures derived from fresh human fecal slurries.^90^ Compared with pH 6.8, a more acidic environment (~pH 6.0) suppressed *Bacteroides* growth and promoted the proliferation of *Lactobacilli.*^90^ Amplicon (16S ribosomal RNA gene) sequencing further demonstrated that pH is a major determinant of microbial community structure in human fecal enrichment cultures *in vitro.*^91^ In the weighted UniFrac analysis, culture pH explained 67% of the microbiome variance in the first principal component, with this variation primarily driven by an expansion of *Lactobacillales* and a reduction in *Bacteroidales* and *Enterobacteriales.*^91^ Moreover, anaerobic infections in the colon are induced mainly by *Clostridia* species, and alkaline stool pH is correlated with *Clostridiodes difficile* infection.^92^ Similarly, *in vitro* human gut microbiota cultures treated with acidic pH-adjusted growth media significantly reduce the overall proportion of *Clostridia* species.^49^^,^^91^^,^^93^ In parallel, acidic pH promotes the abundance of beneficial bacteria and influences not only the composition, but also the metabolic activity and functional output of the gut microbiota, particularly through the fermentation of dietary macronutrients.^39^

### Colonic pH and microbiota‒diet interactions

Gut microbial composition is highly heterogeneous between individuals, with diet accounting for over 50% of microbial variations in mouse models and approximately 20% in humans.^94-96^ Thus, short- and long-term diet modifications have considerable impacts on shaping the gut microbiota.^97^ For example, mice switched from a low-fat diet, enriched with plant polysaccharides, to a high-fat Western diet, low in plant polysaccharides, showed changes in microbial composition and metabolic pathways after just 1 d.^35^ Human studies have also shown remarkable differences in gut microbial composition based on population groups and distinct dietary patterns.^98^ A landmark paper showed that the bacterial profiles of children from Africa, who consumed a diet high in fiber, had higher abundances of metabolite-producing bacteria than European children, who consumed a typical Western diet.^98^ Building on these insights, it is now well-understood that the amount and type of dietary macronutrients, particularly complex carbohydrates, profoundly influence the gut environment, including the gut microbiota, intestinal transit time, and, therefore, pH.^99^ Specifically, the by-products released from their microbial degradation play a significant role in colonic pH.^86^ The main carbohydrates available to bacteria inhabiting the colon are fermentable fibers, such as resistant starches, inulin-type fructans, and galacto-oligosaccharides.^40^ This is because the host lacks the enzymatic capacity to degrade structural polysaccharides and, as a result, they bypass the small intestine and are subsequently fermented by the colonic bacteria.^100^^,^^101^ The main products of this process are SCFAs, particularly acetate, butyrate and propionate, typically in a 3:1:1 ratio.^88^^,^^102^^,^^103^ With pKa values between 4.7 and 4.9, SCFAs predominantly exist in their ionised forms and act as major proton donors within the colonic lumen.^104^ The concentrations of SCFAs in the proximal colon, which has the highest microbial abundance and activity in the gut, are exceedingly high—up to 130 mmol/kg in humans^88^—therefore, it is undeniable that they are key contributors to colonic pH (Figure 2). Indeed, SCFA levels in the large intestine and portal vein,^47^ hepatic and peripheral venous blood,^88^ and cecal and fecal samples^87^ are negatively correlated with the colonic luminal pH in both animal models and humans. Importantly, approximately 95% of SCFAs produced by the gut microbiota are rapidly absorbed by colonocytes,^103,105–107^ which may explain why pH gradually increases from the proximal colon to the distal colon, thereby challenging the reliability of fecal pH as a direct proxy of colonic pH or SCFA production. Among these, butyrate is preferentially utilized as an energy source by colonocytes.^108^ Propionate is largely metabolized within the liver, whereas acetate, together with smaller proportions of butyrate and propionate, escapes first-pass metabolism and enters the systemic circulation.^109^

The first study to evaluate how dietary fiber influences luminal and interstitial pH in the large intestine, currently available as a preprint, demonstrated that the luminal pH of mice fed a control diet (4.7% crude fiber) was approximately 7.2.^47^ Remarkably, a high-fiber diet (9.7% crude fiber; modified resistant starch supplementation) significantly reduced cecal and colonic pH to approximately 6.0 and 6.5, respectively, with a corresponding decrease in interstitial fluid pH.^47^ This acidification coincided with a marked increase in colonic acetate and total SCFA levels (Figure 2). In contrast, mice fed a low-fiber diet exhibited colonic luminal pH levels of ~7.5 and reduced SCFA concentrations.^47^ These effects were abolished with an antibiotic cocktail.^47^ The high-fiber diet enriched SCFA-producing taxa such as the *Lachnospiraceae-NK4A136* group.^47^ In contrast, the low-fiber diet increased *Alistipes*, a genus previously linked to intestinal inflammation and hypertension.^28^^,^^35^ Notably, SCFA levels in the peripheral circulation remained unchanged following the 7-d intervention,^47^ suggesting that fiber intake primarily exerts its effects locally within the colon.

Aligned with the impact of pH on the composition of the gut microbiota, pH profoundly regulates the metabolic activity of the gut microbiota. *In vitro* cultures of the human gut microbiota typically produce the highest total SCFA levels at pH 6.0, which reflects the physiological pH of the proximal colon.^110^ As the most favorable energy source of colonocytes, butyrate production is significantly enhanced under more acidic conditions (pH 5.5–6.0), and butyrate-producing bacteria, such as *Roseburia* spp*., Faecalibacterium prausnitzii, Coprococcus comes* and *Eubacterium rectale,* thrive under such acidic environments.^49^^,^^50^^,^^93^^,^^111^ Interestingly, although acetate concentrations progressively declined under these conditions, a marked shift was observed at pH 6.5, where acetate emerged as the predominant SCFA.^50^ Similarly, human fecal slurries obtained from four adult volunteers consuming a Western-style diet were incubated under various pH-adjusted conditions; at pH 5.2, there was a significant reduction in net acetate production.^112^ However, at pH 5.9, acetate formation was restored,^112^ indicating a pH-dependent modulation of microbial acetate synthesis. The pH-sensitivity of acetate production may be explained by the predominance of acetate-producing bacteria within the *Bacteroidetes* phylum,^113^ whereby an *in vitro* study demonstrated that the abundances of *Bacteroides* spp. are lower and their metabolic activity is also reduced at pH levels of 5.5 and below.^114^ Another study reported that pH values above 7.0 led to lower butyrate production, and *F. prausnitzii* and *Coprococcus* became highly sensitive to pH environments greater than 7.5.^93^ Interestingly, between pH 6.0 and 7.0, propionate production exceeds that of butyrate. In particular, propionate-producing bacteria such as *Veillonella* spp., *Megasphaera elsdenii* and certain *Bacteroidetes* spp. (e.g., *B. fragilis* and *B. ovatus*) emerge as the dominant carbohydrate fermenter, particularly between pH 6.5 and 7.0.^50^^,^^93^^,^^111^ However, similar to butyrate, propionate accumulation was lower when pH values exceeded 7.0, and this was accompanied by a decrease in the abundance of propionate-producing bacteria.^93^ Acetate, propionate, butyrate, and a combination of all three main SCFAs inhibit the growth of pathogenic *Escherichia coli* (*E. coli*) in a pH-dependent way.^115^ Notably, an SCFA cocktail resembling their ratio in the human colon suppressed the growth of *E. coli* by 96% at pH 6.0, while only 2% inhibition was observed at pH 7.0.^115^

While SCFAs are the most abundant microbial acids and, therefore, the dominant contributors to colonic acidification, a variety of other microbial and host‑derived metabolites also influence the luminal pH.^116^ The combined action of these acidifying and alkalinizing factors establishes a dynamic equilibrium within the colonic environment. For example, lactate is another by-product of fermentable carbohydrates, rather than from direct glucose breakdown in the small intestine.^112^^,^^117^^,^^118^ Lactate is an important intermediate microbial metabolite that can be further metabolized by cross-feeding bacteria to produce SCFAs.^117^^,^^119^ Lactate is typically present at low concentrations in the colon (<3 mM)^88^^,^^120^ due to optimal metabolism occurring at pH values as low as 5.9.^112^ For example, *in vitro* studies using human gut microbiota demonstrated that lactate accumulation rarely occurs when pH exceeds 6.0.^50^^,^^93^^,^^111^^,^^112^ Acidic conditions below pH 6.0, however, favor the growth of lactate-producing bacteria, particularly common probiotic genera such as *Bifidobacterium* and *Lactobacillus*, which belong to the phylum *Actinobacteria.*^50^^,^^93^^,^^111^ Indeed, culturing at pH 5.5 expands *Actinobacteria* populations compared to more neutral pH levels.^110^^,^^121^ In contrast, low pH generally inhibits lactate-utilizing bacteria, such as *Veillonella* spp. and *Eubacterium hallii*, leading to lactate accumulation.^110^ On the other hand, the microbial fermentation of protein, as well as urea metabolism, leads to the production of ammonia.^122-124^ The pKa of ammonia is 9.02; therefore, it is considered a proton recipient and mainly presents as NH_4_^+^ within the colonic lumen.^125^ Although ammonia is theoretically expected to increase the colonic pH, its contribution appears minimal, most likely due to the buffering capacity of abundant SCFAs. For example, protein supplementation markedly increased fecal ammonia levels but did not significantly affect SCFA production or colonic pH.^126-128^ Thus, while fecal ammonia increases with protein supplementation, its transient presence or reduced efficacy in the colon may limit its impact on colonic pH.

In summary, the presence or absence of fermentable dietary fiber and the resulting levels of SCFA production appear to be the primary determinant of colonic luminal pH. In turn, this acidic environment favors the growth of beneficial SCFA-producing bacterial species, creating a feed-forward cycle that overrides the more transient effects of other diet-derived metabolites.^129^^,^^130^ Thus, it is challenging to consider colonic pH independently of, or not accounting for, the profound effects of these metabolites. It is also important to consider that excessive intake of fermentable dietary fiber may also result in SCFA accumulation to concentrations that could inhibit further microbial fermentation. This may occur through several mechanisms, including end-product inhibition, where high SCFA levels lower the intracellular pH and disrupt microbial enzyme activity,^131^^,^^132^ as well as substrate saturation,^133^^,^^134^ wherein the microbial community's fermentative capacity becomes limited despite continued substrate availability. Additionally, elevated SCFA concentrations can reduce bacterial growth rates and shift community composition,^135-137^ potentially decreasing the efficiency of fiber fermentation over time.

## The role of pH in hypertension pathophysiology

Current evidence demonstrates that deviations in systemic and cellular pH can significantly influence cardiovascular health by affecting processes such as cellular metabolism.^138^ For example, disruptions in these acid–base regulatory pathways have been observed in individuals with hypertension, who exhibit greater impairments in acid–base transport function.^139^ Cardiac ischemia (i.e., the cessation of coronary blood flow) and hypoxia (i.e., limited oxygen availability with sustained blood flow) represent major metabolic stressors for the heart, both of which disrupt normal pH homeostasis and can become detrimental to cellular function.^140^ These disruptions often manifest as metabolic acidosis, which is characterized by arterial blood pH levels of <7.40 and reduced levels of bicarbonates.^141^^,^^142^ Metabolic acidosis typically occurs when the host produces excess amounts of acids, or when the kidneys fail to adequately excrete acid from the body.^143^ Accordingly, it is not surprising that it has been linked to impaired cardiovascular function, as well as altered endocrine and renal function.^144-146^ Studies dating to the late 1980s demonstrated that plasma pH and bicarbonate were lower in spontaneously hypertensive rats than normotensive controls.^147^^,^^148^ Importantly, metabolic acidosis preceded the development of hypertension in these rat models,^147^ suggesting that changes in acid-base homeostasis were not the product of elevated BP; rather, they may actively contribute to the onset and progression of hypertension. Supporting this, Sprague–Dawley rats subjected to a weak acid solution as a model of chronic metabolic acidosis demonstrated a sustained elevation in mean arterial pressure that was significantly associated with reduced blood pH.^143^ A cross-sectional study has also inferred that acute metabolic acidosis and BP may be correlated through elevated serum anion and bicarbonate measures.^149^^,^^150^ However, a key question that remains is how the gut intersects with these systemic pH-regulating mechanisms. While severe systemic acidosis, as observed in models of hypertension, represents a pathological reduction in pH that is harmful, more moderate and localized changes in the colonic luminal pH may operate differently. Strikingly, *in vivo* measurements of the intraluminal pH using a wireless monitoring system (SmartPill™ Motility Testing System) revealed that participants with hypertension have higher colonic minimum pH compared to participants with normal BP.^151^ Therefore, elucidating whether and how these physiological pH shifts extend beyond the GI tract represents a critical step toward understanding how large-intestinal pH environments contribute to host cardiovascular outcomes.

### The interplay between colonic pH, diet, and the gut microbiota in hypertension

Hypertension is not the result of a single pathological process but rather comprises multiple contributing factors, including renal dysfunction, vascular remodeling and inflammation.^152^ Emerging evidence highlights the gut as a critical interface in this complex pathology.^14^ However, the role of the colonic pH in this process remains a novel concept. Fluctuations in colonic pH may be driven by microbial fermentation of dietary fiber and local SCFA accumulation. Thus, while the impact of fiber-derived SCFAs may appear initially localized, their downstream effects on host physiology suggest a broader role in shaping the pathophysiological landscape of hypertension.

Dietary fiber intake has long been recognized as a key modulator of BP, and, in recent years, dietary fiber has garnered increasing attention for its consistent cardioprotective effects in the context of hypertension.^153^^,^^154^ A meta-analysis published in 2019, which included 185 prospective studies involving ~135 million person-years and 58 clinical trials involving 4635 adult participants, robustly demonstrated the strong association of dietary fiber intake and BP regulation.^38^ Another meta-analysis, including 12 clinical trials involving 878 adults with CVD or hypertension, reported strong evidence showing that increasing daily fiber intake by 5 g reduces systolic BP by 2.8 mmHg and diastolic BP by 2.1 mmHg.^155^ Notably, dietary fiber is the principal substrate for SCFA production in the colon, and all evidence suggests that SCFAs mediate the cardioprotective effects of a high-fiber diet.

Dietary supplementation with a high-fiber diet or direct acetate administration via drinking water, initiated three weeks before hypertensive induction and continued for six weeks post-surgery, significantly reduced BP by 35% in the deoxycorticosterone acetate (DOCA)-salt mouse model of hypertension.^45^ Similarly, mice receiving a slow-pressor dose of Ang II over four weeks, alongside a low-prebiotic fiber diet supplemented with SCFAs in drinking water, also showed reduced BP, with acetate exerting the strongest antihypertensive effect.^43^ Importantly, acetate modulated the gut microbiota and increased the prevalence of acetate-producing bacteria, predominantly *Bacteroides acidifaciens,*^45^ a species within the *Bacteroides* genus that thrives at pH 6.7,^49^ which is physiologically similar to the colonic luminal pH following a high-fiber diet.^47^ These changes were accompanied by improved cardiac and renal function (i.e., decreased fibrosis and cardiac hypertrophy) and downregulation of pro-inflammatory cytokine signaling in the kidney.^45^ Several independent studies have also confirmed that chronic delivery of all three SCFAs in experimental models of hypertension, either via the drinking water or intraperitoneal injections, lowered BP.^28^^,^^43^^,^^44^^,^^156^ More recently, these findings were clinically validated in a randomized, double-blind, crossover clinical trial (*n* = 20), in which colonic delivery of acetate- and butyrate-enriched resistant starch lowered 24-h systolic BP in patients untreated for hypertension by 6.1 mmHg.^46^ This was associated with an expansion of fecal bacteria that produce SCFAs, 7.8-times higher acetate and butyrate, and a significant decrease in colonic pH (measured using the SmartPill device).^46^ Conversely, a recent double-blind, placebo-controlled, crossover trial in 21 untreated hypertensive participants examined the effects of twice-daily oral sodium butyrate for 4 weeks.^157^ Interestingly, the authors did not observe the anticipated reduction in systolic BP; instead, the butyrate arm showed an average increase of approximately 10 mmHg in systolic and 5 mmHg in diastolic BP compared to the placebo arm.^157^ There are remarkable differences in these two trials, discussed in detail elsewhere.^158^ Importantly, sodium butyrate is readily absorbed in the small intestine rather than reaching the colon,^159^ suggesting that the observed effects are likely independent of colonic-derived butyrate^158^ and its role in lowering colonic pH. Nonetheless, colonic pH may represent a critical intermediate link between dietary fiber, SCFAs, and BP regulation. G protein-coupled receptors (GPCRs) are increasingly recognized as key molecular mediators of gut–host communication.^160^^,^^161^ Proton-sensing GPCRs, which are activated by changes in extracellular pH, may play a central role in how colonic pH regulates BP.

## pH-sensing G protein-coupled receptors

SCFAs, the primary drivers of changes in intestinal pH, are known to act via SCFA-sensing GPCRs, such as free fatty acid receptor 2 (FFA2) and free fatty acid receptor 3 (FFA3) (formally GPR43 and GPR41, respectively).^162^^,^^163^ These receptors are essential for facilitating the signaling between SCFAs and BP-lowering responses in animal models and humans.^160^^,^^161^^,^^164-166^ Since circulating SCFAs are present at low concentrations and primarily act through their canonical receptors, their ability to lower intestinal pH suggests that some effects may be indirectly mediated via pH-sensing receptors. Importantly, the primary ligand of these receptors are protons; that is, they are directly activated by extracellular acidic pH. Their activation is therefore not due to SCFAs themselves, but rather to the increased proton concentration generated by SCFA‑induced acidification of the colonic lumen. Luminal pH has been implicated in modulating colonic epithelial functions, including sodium and water absorption, as well as the uptake of bacterial metabolites by colonocytes.^167^ While this represents an intriguing avenue of investigation, direct evidence remains limited and lies beyond the scope of the present review. Moreover, the roles of acid-sensing ion channels (ASICs) and other pH-sensitive channels have been extensively characterized elsewhere;^168^^,^^169^ this review focuses specifically on GPCRs as key mediators of pH-driven signaling. Understanding how intestinal pH influences pathophysiological processes such as hypertension by activating these receptors will help reveal largely unknown gene-by-environment interactions, offering opportunities for innovative therapeutic strategies.

**Figure 3.**
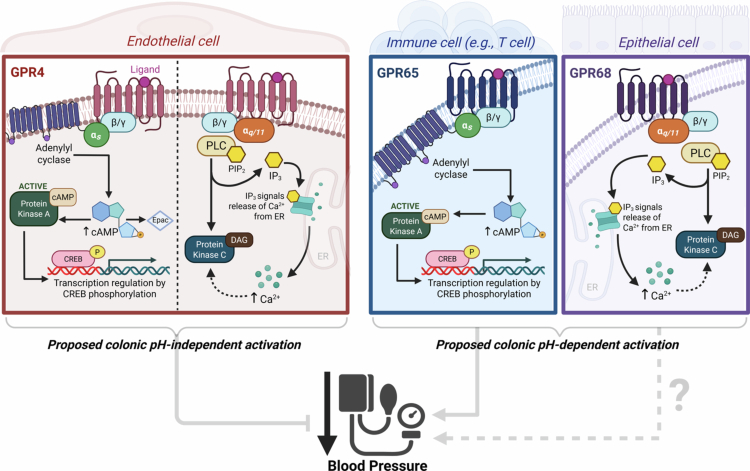
Downstream signaling pathways of the pH-sensing GPCRs GPR4, GPR65, and GPR68 in distinct cell types. GPR4 is expressed primarily in endothelial cells and signals through both Gα_s_ and Gα_q/11_ pathways, leading to increased intracellular cyclic adenosine monophosphate (cAMP) and activation of the phospholipase C (PLC) signaling pathway, respectively. Increased cAMP levels activate the enzyme protein kinase A, leading to the phosphorylation of cAMP response element-binding protein (CREB). CREB is a transcription factor that binds to cAMP response elements in DNA to regulate the transcription of target genes. cAMP also leads to increased levels of exchange protein directly activated by cAMP (Epac). Epac is an effector protein of cAMP that is involved in a number of PKA-independent processes.^181-183^ PLC is activated by phosphatidylinositol 4,5-bisphosphate (PIP_2_) and leads to the production of inositol 1,4,5-trisphosphate (IP_3_). IP_3_ accumulation signals calcium (Ca^2+^) release from the endoplasmic reticulum (ER), leading to intracellular Ca^2+^ accumulation. Both Ca^2+^ and diacylglycerol (DAG) can activate the enzyme protein kinase C (PKC), which, in turn, leads to the phosphorylation of target proteins; however, the relevance of this mechanism in the context of blood pressure regulation remains unclear. GPR65 and GPR68 are predominantly expressed in immune cells (e.g., T cells); GPR65 preferentially couples to the Gα_s_ pathway, while GPR68 signals via Gα_q/11_. However, it has been proposed that only these two receptor-specific pathways may have distinct roles in colonic pH-dependent vascular and immune responses. Figure created with Biorender.com.

The family of proton-sensing GPCRs, including GPR4, T cell death-associated gene 8 (TDAG8/GPR65) and ovarian cancer G protein-coupled receptor 1 (OGR1/GPR68), was first described by Ludwig et al. in 2003 (Figure 3).^170^ A fourth receptor, G2A (GPR132), was also identified; however, because this receptor family has attracted more attention, the role of GPR132 as a proton sensor has become more controversial than that of the other three receptors.^171^ Amino acid sequencing analysis, site-directed mutagenesis, and high-resolution cryo-electron microscopy revealed that specific crucial histidine residues in their extracellular domain are responsible for detecting increased extracellular proton concentrations and subsequent activation.^170^^,^^172^ Proton detection can only occur in the presence of five amino acids: aspartic acid, glutamic acid, histidine, arginine, and lysine.^173^ While pH sensing occurs via electrostatic interactions with aspartic and glutamic acid residues, it is the histidine groups that are proposed to act as proton-sensing. More specifically, protonation of the histidine imidazole groups disrupts existing hydrogen bonds between these residues, leading to a conformational change in the receptor's active state.^170^ The imidazole side chain of histidine appears to serve as a precise proton receptor through its pH-dependent charge distribution,^174-176^ highlighting the importance of histidine in pH-dependent activation by these receptors. Interestingly, however, these histidine residues are not conserved among the four receptors; GPR4 and GPR68 have the strongest amino acid similarity, whereas GPR65 differs in both its amino acid sequence and histidine distribution.^171^^,^^177^ GPR132 is the most architecturally distant from the other receptors, most likely owing to the lack of critical histidine residues at the corresponding positions.^178^ Indeed, more recently, it has been reported that the amino acid triad, comprising a pair of closely spaced aspartic acid residues and glutamic acid, is conserved in GPR4, GPR65, and GPR68 but not in GPR132, which may also explain its weaker proton-sensing and activation.^173^ In addition to GPR132's relatively weak activation by protons, it has been reported that its primary activation may occur through oxidised fatty acid derivatives.^179^^,^^180^ Notably, its maximal activation has been reported to occur around pH 7.4,^171^ thereby limiting its physiological relevance in the acidic environment of the colonic lumen, where SCFA-mediated pH reductions are most pronounced. Therefore, for the remainder of this review, we focus exclusively on the proton-sensing receptors GPR4, GPR65 and GPR68.

All three receptors exhibit maximal activation around pH 6.6 ± 1.0 and are largely inactive at alkaline pH values (7.6–7.8),^170^^,^^177^^,^^184^ responding to mildly acidic extracellular conditions that are physiologically relevant. Proton binding causes receptor conformational change and the subsequent activation of the intracellular heterotrimeric G protein. Once activated, GDP is released from the G protein and replaced by GTP, leading to the dissociation of the Gα subunit and the Gβγ dimer. This unmasks interactive domains that can modulate a variety of signal transduction pathways through activation of effector proteins, including adenylyl cyclase, ion channels and phospholipase C.^172^^,^^185^^,^^186^ The coupling preferences for each receptor can vary depending on specific physiologies and cell background.^187^^,^^188^ Several studies have reported that these proton-sensing receptors mediate cellular actions in various cell types, as discussed in more detail below.^170^^,^^189^ Since their discovery, these receptors have been implicated in multiple biological processes and disorders, including ischemia, inflammation and tumor microenvironments.^190-196^ However, despite these insights, these receptors are still classed as orphan receptors with much to learn about their unique pharmacology.

### GPR4

GPR4 is predominantly expressed in vascular-rich tissues, including vascular endothelial cells,^197^^,^^198^ the kidney cortex, isolated kidney collecting ducts, and the inner and outer medulla.^199^ Moreover, ubiquitous expression has also been reported in organs including the heart, lungs, liver, white adipose tissue and neurons of the retro-trapezoid nucleus.^181-183^^,^^199^ GPR4 exhibits a distinct but relatively broad activation profile, responding to pH changes between 5.8 and 7.6.^170^^,^^171^^,^^200^ A mutational study demonstrated that each of the histidine residues, His-79, -165 and -269, is required for proton-dependent activation.^188^ More recently, high-resolution cryo-electron microscopy structures of GPR4 in complex with G proteins have provided further critical insights into the molecular mechanisms of receptor activation under acidic conditions.^172^ You et al. reveal a highly acidic, hydrophilic extracellular domain (ECD) containing the key histidine residues (His-10, -79, -165, -269), cooperatively organized for proton-sensing and activation.^172^ Upon activation, GPR4 can transduce downstream signaling through Gα_s_/cyclic adenosine monophosphate (cAMP) or Gα_q_/phospholipase C (PLC) pathways.^188^^,^^200^^,^^201^ cAMP is a ubiquitous second messenger that controls a wide range of cellular processes mainly through the downstream effector protein kinase A (PKA).^202^^,^^203^ The activation of PKA by cAMP leads to the phosphorylation of a wide array of proteins, altering their function to regulate processes such as gene transcription, cell metabolism, proliferation and differentiation, and ion channel activity (Figure 3).^204^

Specifically, GPR4 contributes to inflammation and leukocyte adhesion in endothelial cells via the Gα_s_/cAMP pathway, mediated by Exchange protein activated by cAMP (Epac) (Figure 3).^197^ Epac is recognized as an effector protein of cAMP, involved in many important cellular processes, including cell adhesion,^205^^,^^206^ as well as the regulation of several PKA-independent processes.^207-209^ Moreover, extracellular acidosis activates GPR4 in endothelial cells. It leads to cAMP accumulation, subsequently inhibiting endothelial cell migration and microvessel growth,^201^ confirming its functional role in endothelial cells and, more broadly, vascular integrity. In a subsequent study, the same authors proposed that GPR4 mediates the response of vascular endothelial cells to acidic tissue microenvironments by triggering inflammatory responses, particularly via upregulation of adhesion molecules and leukocyte binding.^197^ Overall, owing to its high expression level in vascular endothelial cells, GPR4 has been reported to be a regulator of blood vessel stability.^201^ More recently, studies have shown that GPR4 deficiency reduces intestinal inflammation in a murine model of colitis and inflammatory bowel disease (IBD).^191^^,^^210^^,^^211^ Moreover, GPR4 appears to play a paradoxical role in tumor biology; its overexpression in human tumor tissue,^196^^,^^212^^,^^213^ along with its promotion of pathological angiogenesis and tumor growth,^198^ suggests a pro-oncogenic function. On the other hand, GPR4 reduces the migration and metastasis of mouse melanoma and prostate cells *in vitro.*^214^

### GPR65

GPR65 is expressed almost exclusively in lymphoid tissue such as the spleen, lymph nodes, thymus, and bone marrow, owing to its predominant expression in immune cells, including myeloid and lymphoid cells.^215-217^ Maximal activation of GPR65 occurs between pH 6.2 and 6.8, although the receptor remains responsive at extracellular pH levels below 7.4.^177^^,^^218^ Like GPR4, GPR65 preferentially couples with Gα_s_ proteins to activate the cAMP signaling pathway, leading to phosphorylation of the transcription factor cAMP response element binding protein (CREB) and subsequent downstream signaling that modulates the expression of inflammation-related genes.^219^^,^^220^ One such interaction involves binding to the nuclear factor (NF)-κB complex, a key regulator of proinflammatory responses, helping to suppress excessive inflammation (Figure 3).^219^ GPR65 was first identified as a receptor during thymocyte apoptosis.^221^ Although GPR65 deficiency has been shown not to significantly affect thymocyte apoptotic pathways, its activation plays an integral role in T cell function and modulation.^216^ For example, GPR65 overexpression in cultured human CD4+ T cells prompted the polarization of pro-inflammatory T cell phenotypes.^222^ Moreover, mouse peritoneal macrophages exposed to acidic extracellular pH (6.2–7.6) exhibited GPR65-dependent activation, leading to reduced production of inflammatory cytokines TNF-α and IL-6 in response to lipopolysaccharide (LPS) stimulation.^223^ LPS, a component of the outer membrane of Gram-negative bacteria, is well known for its potent endotoxic properties.^224^ In an experimental model of LPS-induced acute lung injury, GPR65 deficiency in mice led to exacerbated phenotypes and worsened outcomes.^225^

GPR65 has been extensively implicated in inflammatory conditions, particularly IBD.^192^^,^^195^^,^^226^^,^^227^ In fact, experimental murine models of IBD have consistently shown that GPR65 deficiency resulted in more severe inflammatory phenotypes, including increased infiltration of macrophages and neutrophils during colonic inflammation.^226^ Notably, GPR65 expression was upregulated in inflamed intestinal tissues of individuals with inflammatory bowel disease.^192^^,^^226^ Macrophages lacking GPR65 exhibited distinct transcriptomic responses to mildly acidic conditions (pH 6.8) compared to wild-type cells, indicating that GPR65 plays a key role in modulating macrophage adaptation to acidosis, which may impact tissue repair and resolution of inflammation.^226^ Importantly, a GPR65 missense variant rs3742704 (I231L) and a GPR65-intronic single-nucleotide polymorphism rs8005161 are among the top variants associated with the risk of human IBD, including ulcerative colitis and Crohn's disease.^227-231^ A phenome-wide association study (PheWAS) across 1228 phenotypes for rs8005161 using data from 647,776 participants from 23andMe confirmed previously reported associations with IBD (specifically Crohn's and ulcerative colitis) and asthma^232^ and identified further associations with eczema (atopic dermatitis).^233^ Furthermore, GPR65 deficiency in mice exacerbated eczematous skin lesions and enhanced leukocyte infiltration in an experimental model of atopic dermatitis.^233^ Another study demonstrated that impaired GPR65 signaling via the cAMP-CREB pathway disrupted the differentiation of T helper-17 and -22 cells by altering lipid metabolism.^234^ Consequently, their impaired differentiation increased susceptibility to both bacterial infection-induced and T cell-mediated colitis.^234^ Moreover, studies have reported GPR65 expression in neurons^183^^,^^235^ and in immune cells localized in the central nervous system,^236^ with growing evidence suggesting that GPR65 is neuroprotective in acute stroke.^190^^,^^237^^,^^238^ Similar to GPR4, GPR65 has both pro- and anti-tumorigenic effects; it is overexpressed in human tumor tissue, particularly in colonic, ovarian, and renal tumors.^196^ However, it has also been proposed as an immune checkpoint in the tumor microenvironment.^239^^,^^240^

### GPR68

GPR68 was first cloned from the HEY human ovarian cancer cell line; hence, it was previously known as the Ovarian cancer G protein-coupled receptor 1 (OGR1). Its proton-sensing role was found to be maximally activated by extracellular protons at pH 6.8, but retains activity across a physiological window extending to pH 7.8.^170^^,^^241^^,^^242^ While histidine residues are critical for activation of this receptor family, mutational studies demonstrated that even in the absence of extracellular histidines, proton-driven stimulation of GPR68 can still occur.^243^ A recent study using in-depth deep mutational scanning involving 9500 mutants determined that GPR68 does not have a single site for proton recognition, instead relies on a network of proton-sensitive residues organized within the extracellular surface and transmembrane domains for activation.^244^ GPR68 expression has been highly associated with immune cells, particularly neutrophils, macrophages, dendritic cells and T cells.^245-247^ Moreover, it is also more widely expressed in cardiovascular-relevant tissues such as the spleen, heart, kidney, and brain.^248^ Specifically, GPR68 is expressed in epithelial cells of the intestine and renal tubules, skeletal myocytes, hepatocytes, peripheral neurons, aortic smooth muscle cells, and cardiomyocytes.^170^^,^^249^ Contrastingly to the other three receptors, GPR68 primarily couples with Gα_q/11_ proteins and activates the phospholipase C (PLC)/calcium (Ca^2+^) signaling pathway, which typically leads to the production of inositol 1,4,5-triphosphate (IP_3_), an important secondary messenger that mediates the release of Ca^2+^ from intracellular stores and regulates many cellular processes, including smooth muscle contraction, endothelial permeability, and immune cell activation and regulation (Figure 3).^170^^,^^250^^,^^251^

In acidic tumor microenvironments, GPR68 has been implicated in regulating phenotypic shifts from M1 to M2 macrophages, with GPR68-deficient mice showing a greater proportion of M1 macrophages in tumors than wild-type mice.^247^ Conversely, GPR68 inhibition was beneficial for tumor suppression by enhancing infiltration of CD8+ T cells, suggesting a dual function.^252^ In a setting of intestinal inflammation, such as a murine model of colitis, GPR68 deficiency was protective against spontaneous inflammation, reported by a significant reduction in rectal prolapse and decreased inflammatory immune cell infiltration.^245^ Similarly, inflamed intestinal samples from IBD patients showed higher GPR68 expression than non-inflamed samples, and both human and murine intestinal fibroblasts exhibited GPR68-dependent activation at acidic pH, suggesting an active role for GPR68 in the pathogenesis of IBD.^193^ GPR68 protein levels were reduced in lung tissues from patients with idiopathic pulmonary fibrosis compared to healthy controls.^253^ This was coupled with GPR68 expression suppressing transforming growth factor-beta (TGF-β)-induced myofibroblast differentiation.^253^ In the context of fibrosis, TGF-β is a key driver of pathological tissue remodeling by promoting the activation and differentiation of fibroblasts into myofibroblasts, which produce excessive extracellular matrix components such as collagen.^254^ Following this, GPR68 was identified as a critical mediator of endothelial dysfunction in *Staphylococcus aureus*-induced lung injury, in which GPR68 activation by an acidic pH stimulus aggravated vascular injury and associated inflammation.^255^ Finally, GPR68 is expressed during early osteoclast development and plays a key role in sensing extracellular acidity in osteoblasts^256^^,^^257^ and, thus, bone density regulation. In GPR68-knockout mice, bone resorption is reduced, and bone density is increased, accompanied by a higher number of osteoblasts and osteoclasts.^258^

## The role of pH-sensing GPCRs in hypertension and their pharmacological potential

The pH threshold for the activation of these receptors is comparable to the luminal pH within the large intestine following a high-fiber diet intervention.^47^ Therefore, when expressed in the intestine or in circulating cells that transit through the gut, such as immune cells, it is plausible that proton-sensing GPCRs contribute to the protective effects of SCFAs. This is particularly relevant given that hypertension is now recognized as an unconventional inflammatory condition, with the immune system playing a central role in both its development and progression.^259^^,^^260^ As such, these receptors may serve as key molecular links between gut-derived metabolites and immune-driven mechanisms underlying BP regulation.

### GPR4 in hypertension

In animal models, hypertension can be pharmacologically induced by administering the peptide hormone Angiotensin II (Ang II).^261^ Ang II is the principal effector of the renin–angiotensin–aldosterone system (RAAS), a key hormonal cascade involved in regulating BP and fluid balance.^262^ Dysregulation of this pathway, particularly through sustained elevation of Ang II, contributes to the development and maintenance of hypertension through chronic vascular resistance and end-organ damage, amongst a range of maladaptive cardiovascular responses.^261^ Interestingly, angiotensin II type 1 receptor (AT_1_) binding was significantly lower in the brains of GPR4-deficient mice compared to their wild-type counterparts;^263^ as this receptor is implicated in the physiological responses to Ang II in the brain,^264-266^ this finding alludes to GPR4 deficiency being protective against Ang II-induced BP elevation. Importantly, GPR4 deficiency led to a 12 ± 2 mmHg reduction in systolic BP compared to wild-type mice.^263^ The kidney, as the central organ of the RAAS,^262^ is also a site of high GPR4 expression.^199^ Genetic deletion of GPR4 has been shown to impair net acid excretion and to induce spontaneous metabolic acidosis,^267^ thereby underscoring its critical role in renal acid–base homeostasis. Collectively, these findings highlight the functional relevance of GPR4 within BP‑regulating tissues and suggest a possible involvement in hypertension pathogenesis.

More recently, a study demonstrated that GPR4 deletion in ApoE-deficient mice significantly attenuated Ang II–induced abdominal aortic aneurysm formation by reducing inflammation, angiogenesis, and endothelial dysfunction via downregulation of transcriptional and angiogenic signaling.^268^ While this work specifically evaluated aneurysm development in the abdominal aorta, it is noteworthy that Ang II is also a well‑established driver of hypertension and vascular pathology in experimental models.^261^ Thus, although direct evidence for hypertensive vascular remodeling is lacking, these findings raise the possibility that GPR4 may contribute more broadly to vascular disease processes and could represent a therapeutic target in abdominal aortic aneurysm, with potential relevance to hypertension. However, in combination with evidence of GPR4 expression, these findings suggest that GPR4 is unlikely to be involved in the colonic pH regulation of BP (Table 1). Thus, GPR65 and GPR68 are the most likely receptors involved in downstream pathways activated by colonic pH that result in changes in BP.

**Table 1.** Evidence for pH‑sensing G protein-coupled receptors in blood pressure regulation and their phenotypes in hypertension and under dietary fiber interventions. Evidence strength is indicated by star ratings (★★★ strong, ★★ moderate, ★ limited). Arrows denote the direction of blood pressure change (↑ increase, ↓ decrease) in whole-body knockout (KO) mouse models, with additional phenotypes listed alongside. Dietary fiber intervention phenotypes are shown as receptor‑dependent effects, highlighting that fiber's BP‑lowering benefits are abolished in KO models.

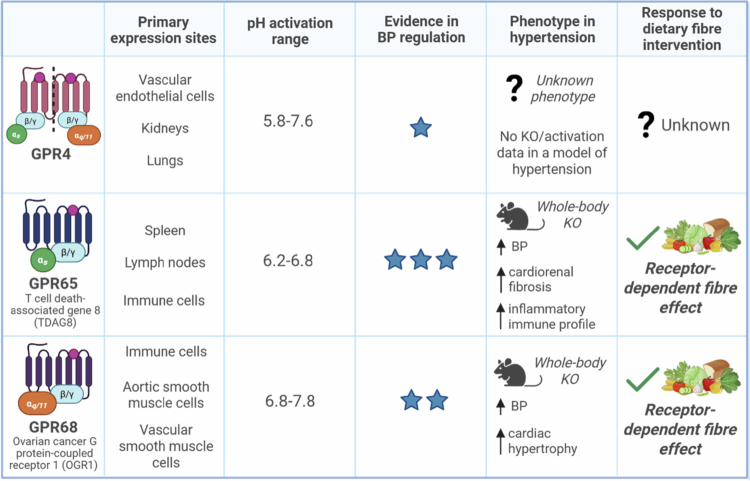

### GPR65 in hypertension

During myocardial infarction, an ischemic heart condition characterized by the occlusion of coronary arteries, GPR65-deficient mice had lower survival rates and worsened cardiac function compared to control mice.^269^ Moreover, *Gpr65* mRNA expression levels were markedly increased in the hearts of mice subjected to myocardial infarction,^269^ highlighting that GPR65-dependent pH changes are physiologically important in ischemic conditions. Though there are currently very few studies investigating the role of GPR65 in BP regulation, a seminal study in the form of a preprint has implicated GPR65 in a murine model of Ang II-induced hypertension.^47^ It was reported that GPR65-deficient mice had significantly higher BP as well as considerably higher cardiac and renal fibrosis compared to hypertensive wild-type mice under a control diet^47^ (Table 1). Importantly, GPR65-deficient mice fed a high-fiber diet had lower BP compared to those fed a low-fiber diet; however, their BP in response to fiber intake was higher than that of wild-type mice,^47^ suggesting that the cardioprotective nature of high-fiber diets and their associated metabolites is partly GPR65-dependent (Table 1).

It is plausible that GPR65 contributes to hypertensive outcomes by regulating the immune system. GPR65 expression in immune cells modulates inflammation in acidic microenvironments through various mechanisms. These include influencing CD4+ T cell differentiation toward Th1 and Th17 cells,^222^ downregulating proinflammatory cytokines such as IL-17A^269^ and, notably, promoting cAMP accumulation and CREB phosphorylation. This latter pathway can suppress key proinflammatory cytokines such as TNFα and IFNγ, both of which are implicated in the pathogenesis of hypertension.^260^^,^^270^ Indeed, GPR65-deficient mice also exhibited a more pronounced inflammatory immune profile in the spleen, including CD8+ and γδ T cell subsets.^47^ Importantly, the authors reported higher GPR65 expression in CD8+ T cells than in CD4+ T cells, with particularly enriched expression in the colonic lamina propria,^47^ suggesting a more prominent role in CD8+ T-cell function in the colon. Functionally, low pH, comparable to that observed in the large intestine following high-fiber dietary intervention, suppressed pro-inflammatory cytokine production by CD8+ T cells from the spleen and colon via GPR65 activation,^47^ providing a plausible mechanism linking dietary fiber, colonic acidification and GPR65 signaling. Supporting a causal role, transfer of *Gpr65*⁻/⁻ CD8+ T cells into *Rag1*⁻/⁻ mice (lacking both T and B cells^271^ led to elevated BP and cardiac hypertrophy under Ang II-induced hypertensive conditions compared to mice receiving wild-type CD8+ T cells.^47^ Together, these findings demonstrate a causal role for GPR65 signaling in CD8+ T cells in BP regulation and the protective effects of a high-fiber diet on cardiovascular complications.

### GPR68 in hypertension

Homeostatic control of vascular resistance and flow-mediated vasodilation are crucial; impairment of these processes is not only a hallmark of endothelial dysfunction but also a precursor to an array of vascular diseases, including hypertension.^272^ A recent study found that, in addition to sensing extracellular protons, GPR68 also has an important role as a mechanosensor of shear stress in human and mouse vascular endothelial cells.^273^ Although no significant differences in BP were observed between GPR68-deficient and control mice, GPR68-deficient mice displayed distinct impairment in flow-mediated vasodilation and remodeling in mesenteric arterioles.^273^ Importantly, GPR68 functions as a mechanosensor by activating the Gα_q/11_/Ca^2+^/IP_3_ signaling cascade,^170^^,^^250^ a pathway now recognized as a crucial signaling component in cardiovascular pathophysiology.^274-277^ Moreover, GPR68 was implicated as a key receptor responsible for extracellular acidic pH-induced production of prostaglandin I_2_, a potent vasodilatory compound, in human aortic smooth muscle cells.^215^ A pivotal study in a rodent model of myocardial infarction reported that GPR68-expressing cardiomyocytes formed a cellular buffer zone around the infarcted myocardium, and upon pharmacological overactivation of GPR68, expression of cardiomyogenic and pro-survival genes was upregulated.^278^ Conversely, a recent study investigating chronic kidney disease-associated cardiac inflammation and fibrosis identified upregulated expression levels of GPR68 in monocyte-derived macrophages, which released significantly higher levels of pro-inflammatory cytokines, including TNF-α and IL-6, when cultured and exposed to acidic conditions.^279^ Given the well-established role of inflammation in the development and progression of hypertension,^259^^,^^260^ these findings suggest that GPR68 may contribute to hypertensive cardiovascular pathology by mediating inflammatory responses in acidified tissue microenvironments.

We recently studied whether GPR68 mediates the BP-lowering effects of dietary fiber using a four-week mouse model of Ang II-induced hypertension^280^ (Table 1). Given its high expression in immune cells, we initially hypothesized that this would occur via an immune-dependent mechanism.^280^ We found that GPR68 was involved in the underlying mechanisms conferring the protective nature of a high-fiber diet and its associated metabolites under hypertensive conditions^280^ (Table 1). Specifically, high-fiber-fed hypertensive GPR68-deficient mice were not protected against Ang II-induced BP elevation or cardiac interstitial collagen deposition and exhibited diminished aortic elastin content Compared to high fiber-fed wild-type hypertensive mice.^280^ This study also reported robust evidence that dietary fiber reduces pro-inflammatory immune cell infiltration into the kidney and thoracic aorta; however, this effect was independent of GPR68.^280^ Moreover, the findings indicated that dietary fiber, rather than GPR68, was the primary driver of changes in large intestinal physiology.^280^ This included a marked reduction in collagen deposition within the muscularis propria and significant shifts in the gut microbiota.^280^ Notably, hypertensive mice fed a high-fiber diet exhibited lower abundances of gram-negative species, such as *Alistipes finegoldii*, which has previously been linked to hypertensive outcomes.^280^

### pH-sensing GPCRs as pharmacological targets

GPCRs encompass the largest membrane protein class in the human genome, comprising over 800 distinct receptors.^185^ They play a crucial role in cellular signaling and are highly valuable to the global pharmaceutical industry. For example, approximately 35% of all FDA-approved drugs act on GPCRs.^186^^,^^281^ Although extensive in number and complexity, GPCRs exhibit a remarkably conserved protein architecture comprising seven transmembrane helices linked by alternating intracellular and extracellular loops.^282^ Extracellular stimuli are transduced into a cellular response via a conformational change of the ligand-bound receptor, which activates the intracellular heterotrimeric G protein.^283-285^ GPCRs are classified into five main families,^286^ with the rhodopsin/β-adrenergic receptor group (family A) representing the largest and most extensively studied.^287^ Notably, pH-sensing GPCRs belong to this family A subclass,^288^ making them promising pharmacological targets for BP modulation, particularly through their roles in immune regulation.

Owing to its prominent role in pro-inflammatory signaling and disease progression, GPR4 has emerged as a key target for the development of small-molecule antagonists. In particular, derivatives of imidazo-pyridine compounds have been identified as novel GPR4 inhibitors,^289-291^ as they specifically target the imidazole side chains of histidine residues, which serve as the primary pH-sensing activation sites.^174^ Several studies have demonstrated that these GPR4 antagonists mitigate inflammatory responses through a variety of mechanisms, such as a reduction in pro-inflammatory cytokine (IL-1 and IL-8), chemokine (CXCL1, CXCL2, CCL2 and CCL7) and adhesion molecule expression (VCAM-1 and ICAM-1) in primary-cultured endothelial cells.^138^^,^^292^ Importantly, IL-1β is a potent pro-inflammatory cytokine of the IL-1 family^293^ and is extensively implicated in the pathogenesis of hypertension.^294^^,^^295^ It has long been known to promote endothelial dysfunction and upregulate adhesion molecules such as VCAM-1 and ICAM-1, facilitating leukocyte infiltration into vascular tissues.^296^ Moreover, the highly selective GPR4 antagonist 13, NE-52-QQ57, reduced intestinal inflammation in a dextran sulfate sodium (DSS)-induced 7-d experimental colitis mouse model.^210^ Specifically, pro-inflammatory cytokines TNF-α and IL-10 expression were significantly reduced in distal colonic tissue of mice treated with the GPR4 antagonist 13 compared with vehicle-treated mice, accompanied by a modest reduction in IL-1β and IL-6.^210^

BTB09089, a small nonpolar organic molecule, has been identified as an allosteric agonist of GPR65, binding to a site distinct from the primary ligand-binding pocket, with emerging evidence supporting its anti-inflammatory potential.^187^^,^^297^ In stimulated peritoneal macrophages isolated from wild-type mice, BTB09089 treatment significantly reduced the production of pro-inflammatory cytokines TNF-*α* and IL-6.^297^ This effect was absent in macrophages derived from GPR65-deficient mice,^297^ suggesting that BTB09089 suppresses inflammatory cytokine production via a GPR65-dependent mechanism of action. Mechanistically, BTB09089 is proposed to enhance GPR65 signaling by increasing intracellular cAMP, which subsequently promotes the phosphorylation of CREB.^219^^,^^220^ Activated CREB exerts broad immunomodulatory effects, including the suppression of TNF-α and IFN-*γ*, which are cytokines known to contribute to the pathogenesis of hypertension.^260^ Supporting this, exposure to low extracellular pH (~6.5), which activates GPR65, has been shown to increase intracellular cAMP levels in splenic cells in a GPR65-dependent manner,^47^ further linking GPR65-induced cAMP production to anti-inflammatory signaling. Moreover, delayed administration of BTB09089 every other day from 3 to 21 d post-ischemic stroke inhibited microglial activation, the resident innate immune cell of the brain, in wild-type, but not GPR65-deficient, mice.^238^ More recently, GPR65-positive allosteric modulators such as BRD5075 and BRD5080 have been shown to enhance GPR65 activation and lower the expression of inflammatory cytokines and chemokines in dendritic cells.^298^ Interestingly, a seminal docking study exploring multiple compounds identified two important compounds, ZINC13684400 as a positive and ZINC62678696 as a negative allosteric modulator of GPR65.^187^ Authors found that ZINC62678696 binds to GPR65 but inhibits its activity, and its treatment suppressed M1 macrophage polarization and reduced the release of inflammatory cytokines including TNF-α, IL-6, and TGF-β in hepatic macrophages.^187^ Although GPR65 has emerged as a promising therapeutic target, notable structural differences exist between the human and mouse GPR65 genes.^187^^,^^298^ Many studies investigating GPR65 agonists have been conducted using the murine receptor, with human GPR65 only recently gaining attention.^238^^,^^297^^,^^298^ As the overarching goal in drug discovery and development is successful translation to the human host,^299^ the potential discrepancies between human and mouse GPR65 make it essential to develop a humanized GPR65 mouse model. This model would not only allow for the evaluation of compound efficacy but also enable exploration of their potential in a whole-body system, as opposed to limiting studies to *in vitro* settings.

Relative to GPR4 and GPR65, pharmacological targeting of GPR68 is still in its infancy. The same study that identified two opposing allosteric compounds for GPR65 also identified that the two benzodiazepines Lorazepam and Ogerin are potent allosteric modulators of GPR68 and are able to shift the H^+^ concentration response profile in a cAMP assay.^187^ Subsequent studies have demonstrated that Lorazepam exerts its effects by inducing Gα_s_ signaling in GPR68-overexpressing HEK293 cells, as well as in primary airway smooth muscle cells and fibroblasts exposed to acidic, pH-adjusted media.^300^ Another agonist, 3,5-disubstituted isoxazoles (Isx), has been shown to activate GPR68, leading to the upregulation of cardiomyogenic and prosurvival genes in subepicardial tissue surrounding the site of myocardial infarction.^278^ Interestingly, both Ogerin and Isx increased the overall number of circulating B cells in wild-type mice,^301^ demonstrating that *in vivo* activation of this receptor can lead to tangible changes in host immune responses. Additionally, authors who first identified Ogerin as a positive allosteric modulator of GPR68 recently conducted a structure–activity relationship study and found that modifying the molecule's benzyl alcohol and benzylamino groups significantly enhanced its activity, resulting in a 33-fold increase in allosteric potency.^302^ Conversely, GPR68 has also been the focus of small-molecule inhibitor discovery efforts. Given its previously reported role in driving intestinal inflammation,^245^ GPR68 inhibition with a novel inhibitor known as GPR68-I significantly ameliorated both acute and chronic DSS-induced colitis in mice.^303^ In a separate study, GPR68 was shown to contribute to cardiac inflammation and fibrosis in a murine model of chronic kidney disease (CKD).^279^ The same research group later demonstrated that *Cephalotaxus harringtonia* var. *nana* extract and its active compound, homoharringtonine, suppressed GPR68 activity, and treatment with these inhibitors significantly attenuated CKD-induced cardiac inflammation, fibrosis, and functional impairment in mice.^304^

## Conclusions

The interplay between colonic pH, the gut microbiota, and host physiology represents a rapidly evolving frontier with profound implications in cardiovascular health. Emerging evidence highlights colonic luminal pH as a dynamic and responsive marker of the gut environment, which is shaped primarily by dietary fiber intake and the subsequent production of SCFAs through microbial fermentation. A more acidic colonic environment not only reflects increased microbial fermentation but also reinforces a favorable microbial composition, establishing a feed-forward loop that enhances gut physiological integrity, immune tolerance, and systemic balance. This gut ecosystem, in turn, has far-reaching effects beyond the GI tract, particularly in modulating BP and inflammation. Alterations in colonic pH and microbiota composition have been increasingly linked to the pathogenesis of hypertension, suggesting that dietary interventions targeting luminal acidification may offer therapeutic benefits. Moreover, the emerging roles of proton-sensing GPCRs, namely, GPR65 and GPR68, as mediators of pH-responsive immune and cardiovascular signaling provide a compelling molecular link between the gut environment and systemic vascular outcomes. Future research should prioritize mechanistic studies that integrate host genetics, microbial function, and pH-responsive signaling pathways to uncover novel therapeutic targets. Clinical translation will also benefit from longitudinal human studies that assess the impact of diet-induced colonic acidification on hypertension. Ultimately, restoring and maintaining a physiologically acidic colonic pH through targeted dietary and microbial strategies and the discovery of novel therapeutics for proton-sensing GPCRs may represent promising avenues for the prevention and management of BP and, more broadly, CVD.

## Data Availability

Not applicable.
